# Quantitative outperforms visual assessment of nonparallel orientation in ultrasound breast imaging reporting and data system

**DOI:** 10.7717/peerj.20992

**Published:** 2026-03-18

**Authors:** Kailiang Chen, Qingfang Chen, Size Wu

**Affiliations:** 1 The First Affiliated Hospital of Hainan Medical University, Haikou, Hainan Province, China; 2 UWE College of Hainan Medical University, Haikou, Hainan Province, China

**Keywords:** Breast, Ultrasound, BI-RADS, Descriptor, Parallel, Nonparallel orientation, Quantitative measurement

## Abstract

**Objective:**

The visual assessment of the “nonparallel orientation” descriptor in the ultrasound Breast Imaging Reporting and Data System (BI-RADS) is subjective and may affect both the accurate interpretation of breast masses and the overall diagnostic performance of the ultrasound BI-RADS. The objective of this study was to determine whether quantitative measurement of the nonparallel orientation descriptor improves diagnostic performance in the evaluation of breast malignancy assessment.

**Methods:**

This prospective study, conducted at a tertiary hospital, analyzed 253 out of 6,893 patients with ultrasound BI-RADS 3-5 solid breast masses. For each mass, parallel or nonparallel orientation was assessed visually, and the orientation angle of breast mass was measured quantitatively on ultrasound image using built-in ultrasound software. Histopathological diagnosis served as the reference standard. Receiver Operating Characteristic (ROC) curve was plotted to determine the optimal cutoff value for the orientation angle in assessing breast malignancy. The diagnostic performances of the standard BI-RADS (using visual nonparallel orientation) and a modified BI-RADS (using the quantitative orientation angle) were compared for malignancy stratification.

**Results:**

McNemar testing demonstrated significant differences in diagnostic outcomes between visual nonparallel orientation assessment and quantitative angle measurement, as well as between the standard and modified ultrasound BI-RADS classifications (all *p* < 0.001). The area under the curve (AUC) for visual assessment was 0.651, compared to 0.838 for the orientation angle (*p* < 0.001). Incorporating orientation angle into ultrasound BI-RADS showed higher diagnostic performance (AUC = 0.922) compared to the standard ultrasound BI-RADS in this cohort (AUC = 0.905, *p* = 0.024).

**Conclusion:**

The quantitative orientation angle is a more reproducible and objective measure. It can serve as a valuable complementary descriptor within ultrasound BI-RADS. Integrating this quantitative angle into a multi-descriptor assessment of breast masses improves the overall diagnostic performance.

## Introduction

Breast cancer is the leading cause of cancer-related mortality in women globally and represents a highly prevalent malignancy (Bray et al., 2024). While no definitive prevention strategy currently exists, early detection through imaging screening and prompt treatment of non-metastatic cases significantly improves survival outcomes, underscoring the critical role of timely diagnosis (Hong & Xu, 2022; Duggan et al., 2020; Pal Choudhury et al., 2020; Velentzis et al., 2023). Mammography and ultrasound serve as primary imaging modalities for breast screening. In Western populations, mammography constitutes the first-line modality, with supplemental ultrasound or magnetic resonance imaging (MRI) employed for diagnostic refinement (Bevers et al., 2018; Wang et al., 2022). In contrast, China’s screening paradigm prioritizes ultrasound due to the high prevalence of dense breast tissue, equipment accessibility, and cost-effectiveness considerations (Sudhir et al., 2021; Liu et al., 2020; Gu et al., 2022; Wang et al., 2015). The American College of Radiology’s 2013 Breast Imaging Reporting and Data System (BI-RADS) 5th edition identifies nonparallel orientation as a malignancy-associated feature of breast masses (D’Orsi et al., 2013). Current ultrasound BI-RADS classifies orientation of breast masses as parallel or nonparallel through visual assessment of ultrasound images, a method inherently limited by subjective interpretation. This limitation is reflected in variable predictive performance across studies: prior investigations (Yoon et al., 2016; Badu-Peprah et al., 2024) demonstrate inconsistent positive predictive value (PPV) and negative predictive value (NPV) when using visual assessment of orientation for malignancy risk stratification.

Emerging evidence suggests quantitative nonparallel orientation of ultrasound BI-RADS descriptor may enhance diagnostic precision (Chen & Wu, 2024). The parallel orientation of ultrasound BI-RADS descriptor is not an appropriate lexicon, for the parallel orientation of the breast mass does not meet the parallel criteria by geometry that a line parallels to the horizon line, and it’s named subjectively according to visual assessment of the images. The previous study found that the orientation of ultrasound BI-RADS descriptor actually associated with the assessment of breast mass is that an orientation with angle between 0° and 22.9° to the horizon line is suggestive benign and an orientation with angle > 22.9° to the horizon line is suggestive malignant (Chen & Wu, 2024). While this quantitative descriptor of orientation angle shows promise for improving malignancy risk evaluation, its clinical utility requires rigorous validation. This study investigates whether quantitative measurement of nonparallel orientation is more appropriate than visual assessment of the nonparallel orientation as an ultrasound BI-RADS descriptor, and compares their diagnostic performance in breast malignancy stratification.

## Patients & Methods

### Study population

**Figure 1:**
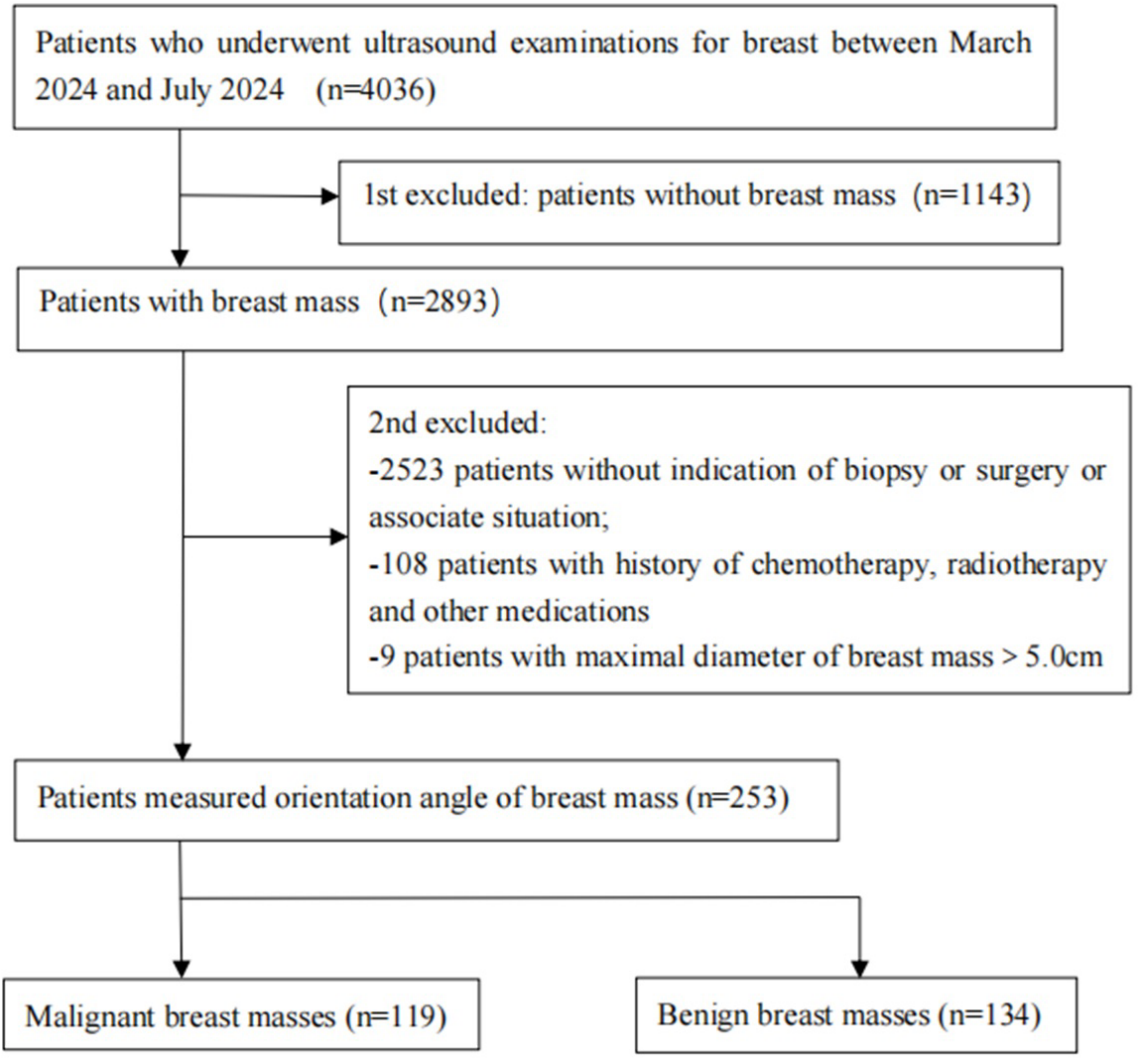
Flowchart showing reasons patients were excluded from this study.

This prospective study was conducted at The Department of Ultrasound, The First Affiliated Hospital of Hainan Medical University from March 2024 to July 2024. The study population involved all consecutive patients who underwent breast ultrasound examinations, with those having breast masses categorized as ultrasound BI-RADS category 3 to 5 considered the target study population. The inclusion criteria were: (1) patients aged ≥ 18 years; (2) patients with breast mass suspicious malignancy or other condition with indication of biopsy or surgery, or intending to undergo self-determined breast surgery or vacuum-assisted breast biopsy; (3) informed consent to participate in the study. The exclusion criteria were: (1) patients without indication of biopsy or surgery or associate situation; (2) patients with a history of neoadjuvant chemotherapy or radiotherapy or other treatment for the target breast mass; (3) patients with breast masses where the maximal diameter > 5.0 cm (unable to fully display the mass in a single scan view). In cases where a patient had more than one breast mass, only the one deemed most suspicious for malignancy or the largest benign mass was included, with the others not measured or included. Figure 1 illustrates the flowchart of the study population selection. Relevant data such as the patient’s age, height, weight, family history of breast cancer, palpability or pain of the breast mass, nipple discharge, skin edema of the breast, and enlarged axillary lymph nodes were recorded.

### Ethical approval

All study-related procedures and data collection were conducted in accordance with the World Medical Association Declaration of Helsinki (revised in 2013). Written informed consent was obtained from all patients.This study was approved by The Ethics Review Committee of The First Affiliated Hospital of Hainan Medical University [2024-KYL-105].

### Ultrasound examination of the breast

The ultrasound examinations were conducted by 16 ultrasound physicians with over 8 years of experience using multi-parameter ultrasound systems including Logiq 9 and Logiq E9 (General Electric Healthcare, Milwaukee, WI, USA), Aloka Prosound α-7 and Aloka Prosound α-10 (Hitachi Aloka Medical Ltd, Tokyo, Japan), Mindray DC 8, Mindray Resona 7, and Mindray Eagus R9S (Shenzhen Mindray Bio-Medical Electronics Co., Ltd., Shenzhen, China), and Philips EPIQ5 (Philips Healthcare, Amsterdam, The Netherlands). Linear array high-frequency transducers were used with frequencies ranging from five to 15 MHz. During the ultrasound examination, patients were positioned lying on the table with their arms raised to fully expose the breast. The upper arms were abducted at a right angle to the body trunk, and the forearms were flexed at a right angle to the upper arms (please refer to the Supplemental Materials S1). An adequate amount of coupling gel was applied to the breast, and the breast was scanned radially from the nipple towards the outer edge in each quadrant to screen and locate any masses. Upon identifying a breast mass, various characteristics such as maximum diameter, position, shape, margin, internal echogenicity, posterior acoustic features, presence of microcalcifications, and edema (peripheral high echoic halo) of the mass were determined through longitudinal, transverse, and oblique multi-sectional scanning. Subsequently, the Color Doppler Flow Imaging (CDFI) mode was activated to detect and evaluate blood vessels within and around the mass. Representative images were stored on the Picture Archiving and Communication System (PACS).

### Measurement of the orientation angle of breast mass

**Figure 2:**
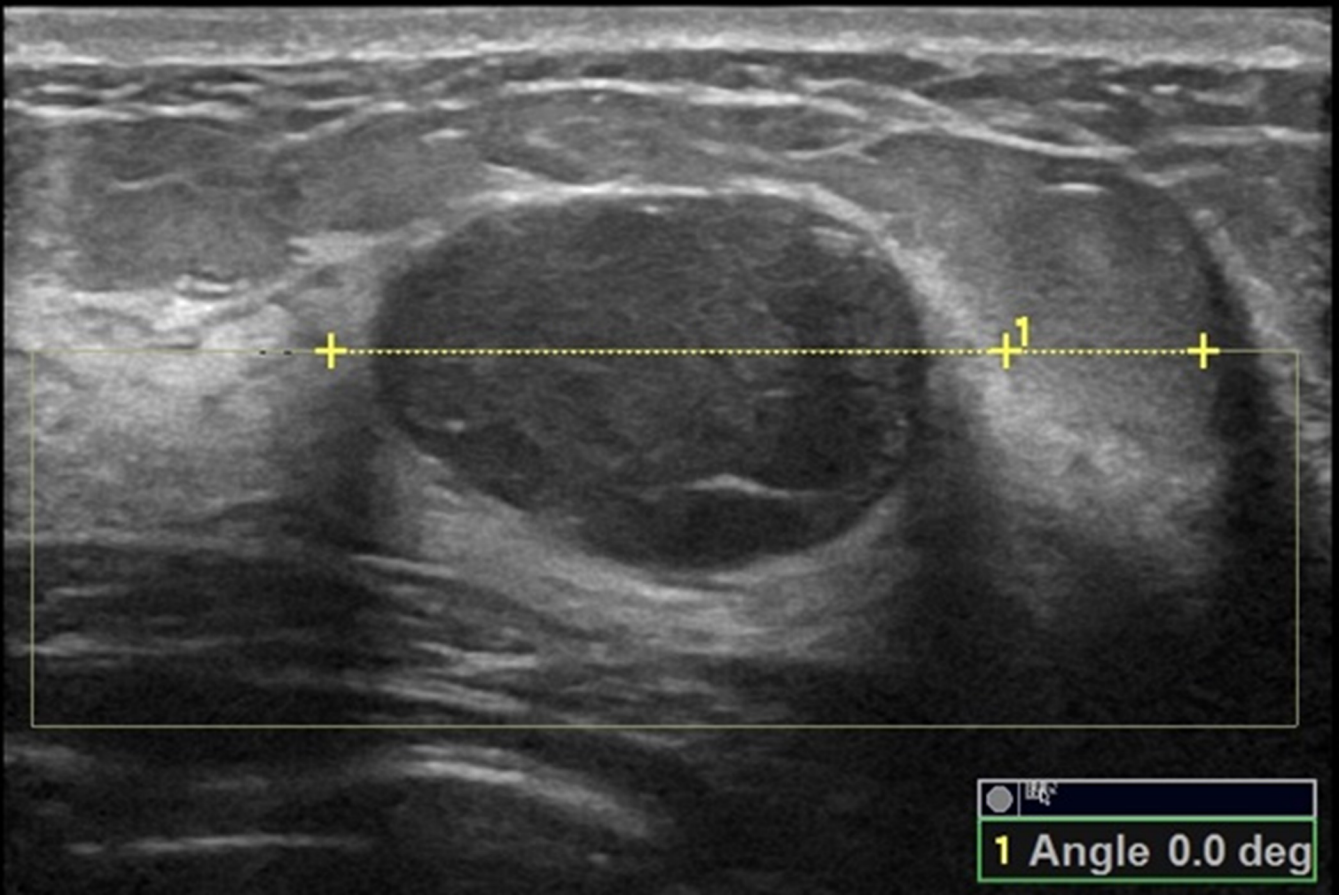
A 22-year-old female with fibroadenoma of the left breast. The orientation angle of the breast fibroadenoma is 0.0 degree. It is categorized as parallel orientation based on visual observation, and it is standard parallel orientation with referring to quantitative measurement.

**Figure 3:**
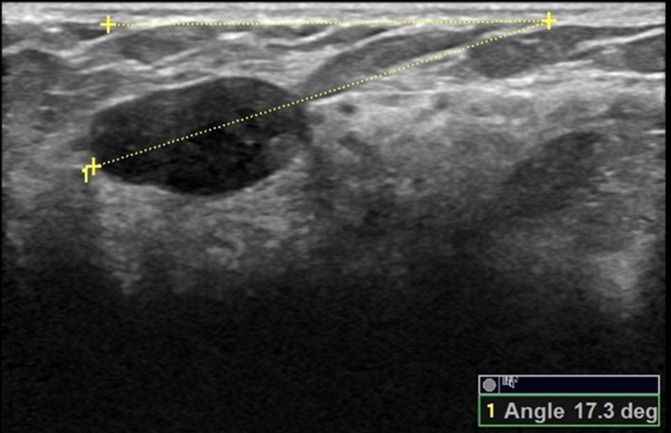
A 30-year-old female with fibroadenoma of the right breast. The orientation angle of the breast fibroadenoma is 17.3 degrees. It is categorized as parallel orientation based on visual assessment, and it is comparable to parallel orientation with referring to quantitative measurement.

**Figure 4:**
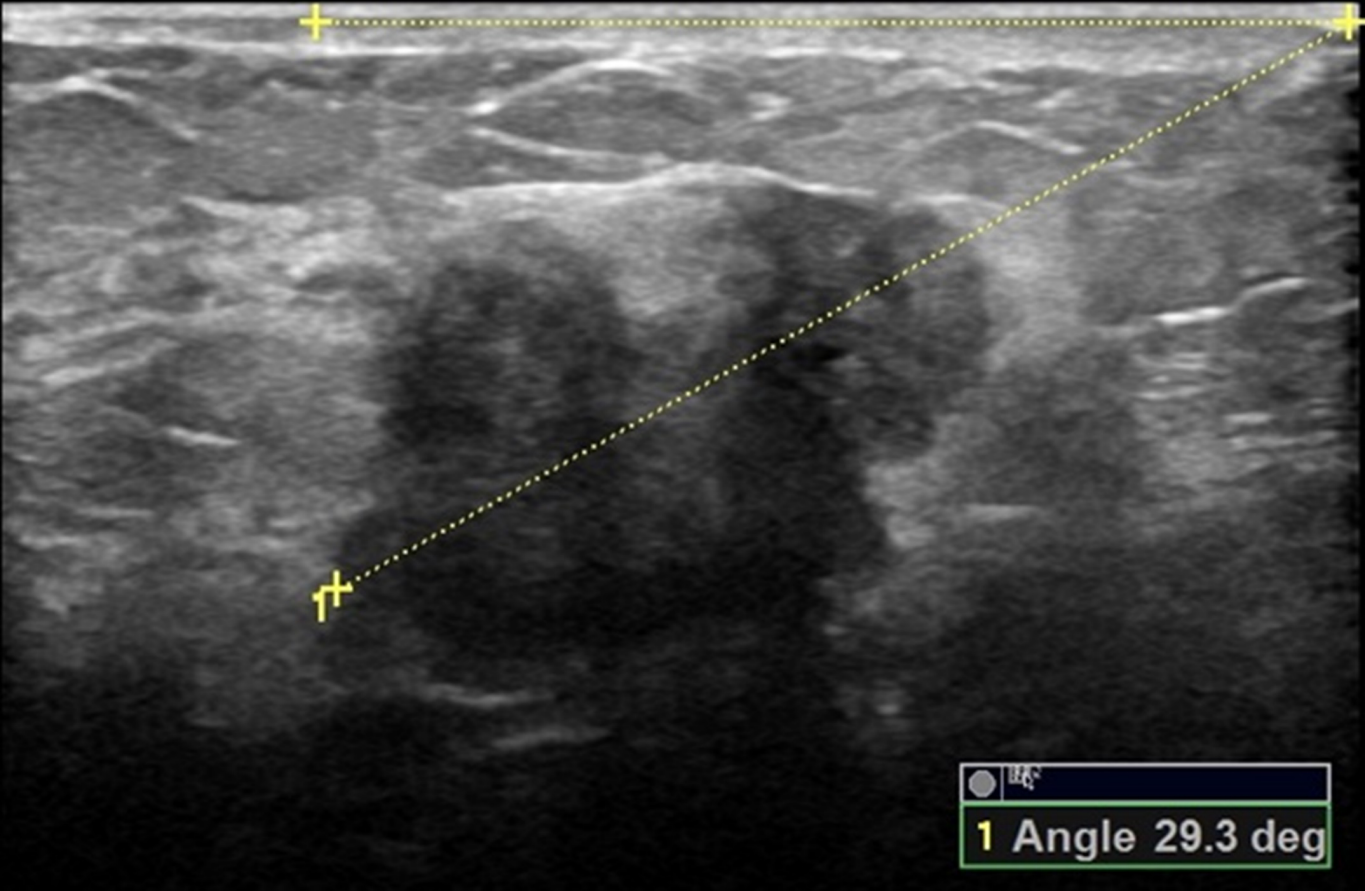
A 57-year-old female with ductal invasive carcinoma of the left breast. The orientation angle of the breast cancer is 29.3 degrees. It is categorized as parallel orientation based on visual assessment, but it is comparable to nonparallel orientation with referring to quantitative measurement.

**Figure 5:**
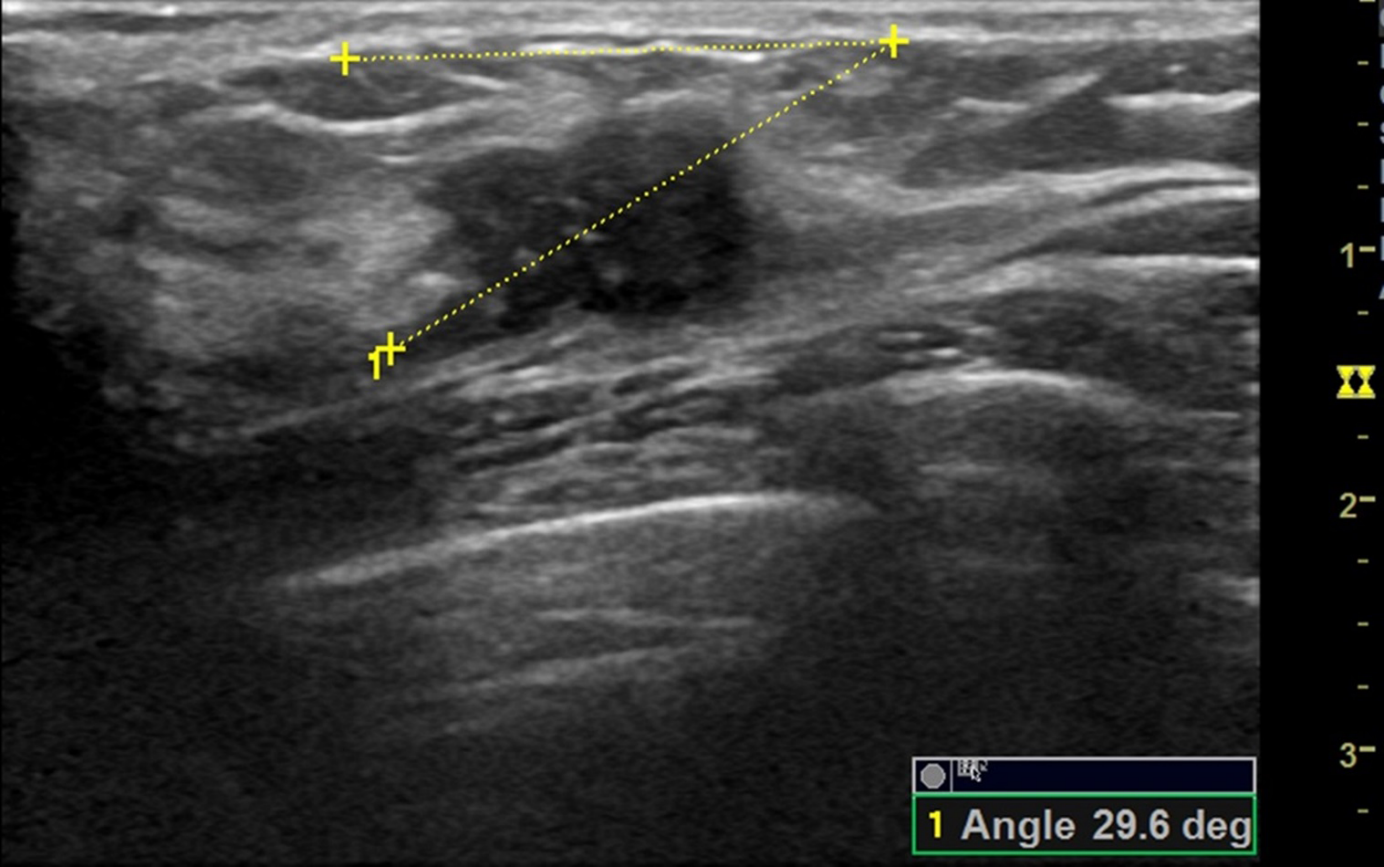
A 42 -year-old female with ductal invasive carcinoma of the right breast. The orientation angle of the breast cancer is 29.6 degrees. It is categorized as parallel orientation based on visual assessment, but it is comparable to nonparallel orientation with referring to quantitative measurement.

**Figure 6:**
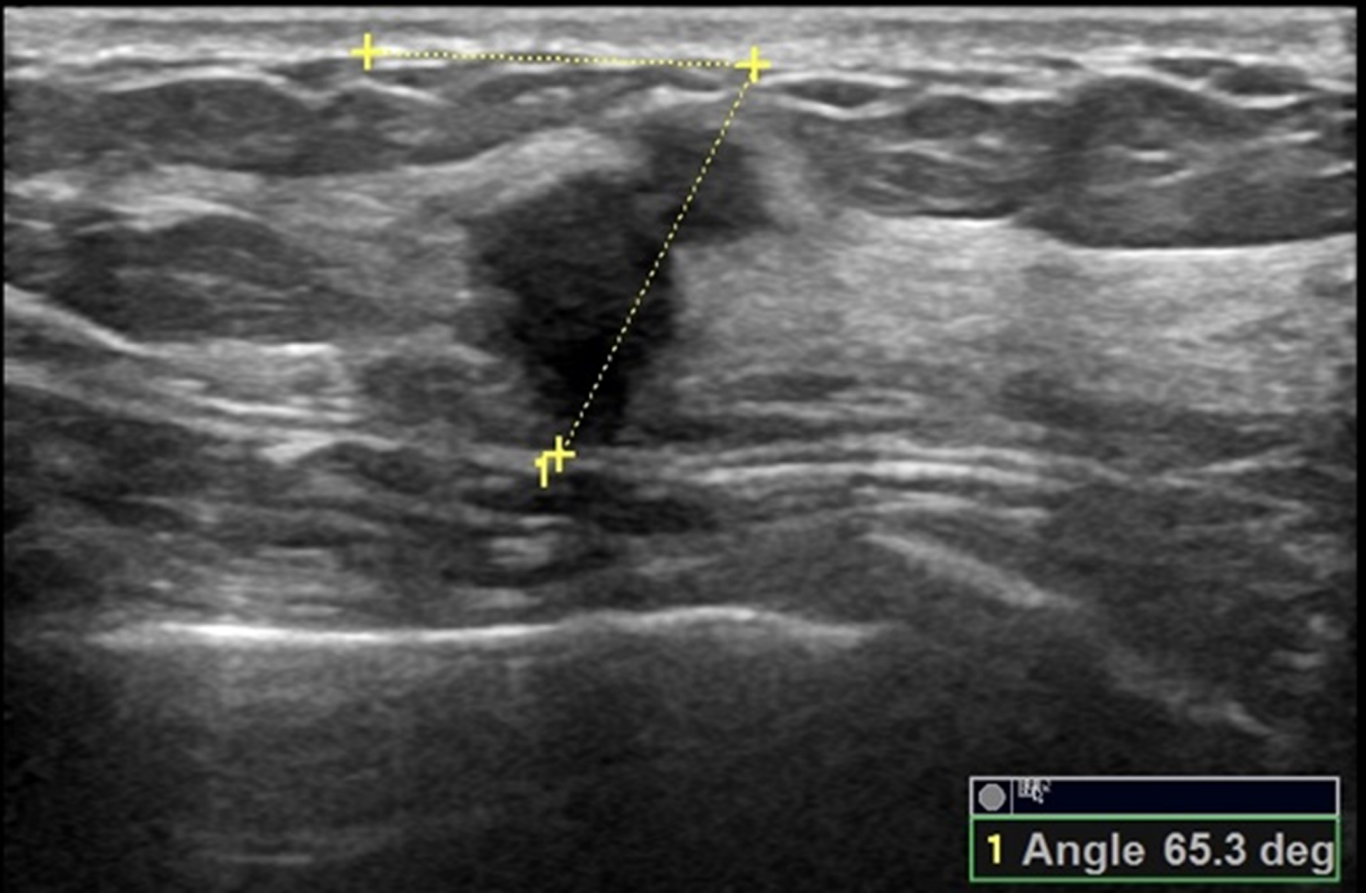
A 58-year-old female with ductal invasive carcinoma of the left breast. The orientation angle of the breast cancer is 65.3 degrees. It is categorized as nonparallel orientation based on visual assessment, and it is comparable to nonparallel orientation with referring to quantitative measurement.

**Figure 7:**
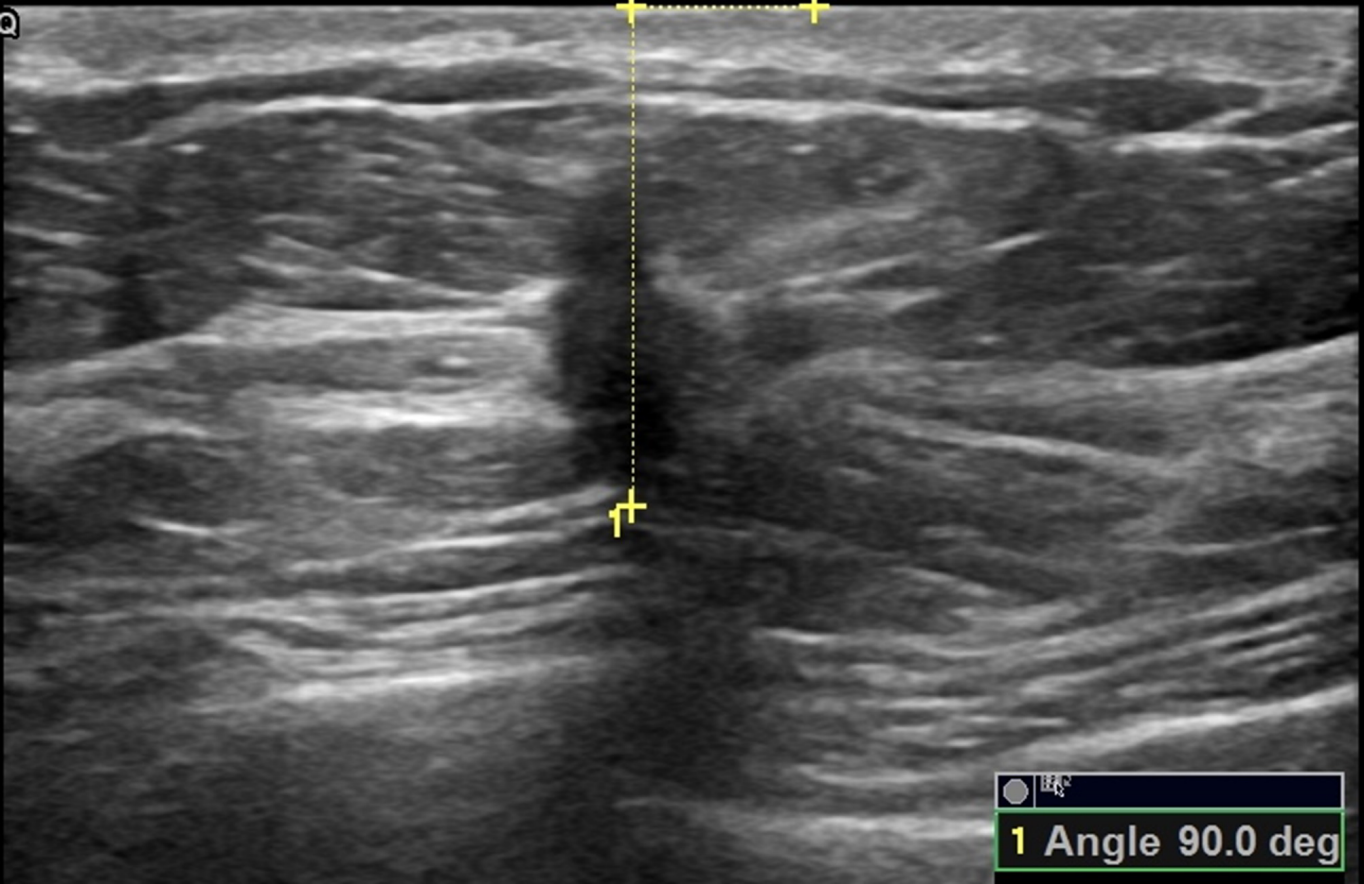
A 43-year-old female with ductal invasive carcinoma of the right breast. The orientation angle of the breast cancer is 90.0 degrees. It is categorized as nonparallel orientation based on visual assessment, and it is comparable to nonparallel orientation with referring to quantitative measurement.

The orientation angle of the breast mass was measured using Logiq E9 and Philips EPIQ5 ultrasound systems by two ultrasound physicians (C. K and W. S) with over 16 years of experience, with the protocol described in a previous study (Chen & Wu, 2024): At first, the breast mass was scanned from different directions, displayed the greatest dimension and frozen the image, then started the angle measurement button of the built-in software of the ultrasound system, drew the line on the maximal longitudinal diameter of the breast mass and extended it to the surface of the skin, drew a line above the breast mass and parallel to the surface of the breast skin and extended it to meet the extension line of the maximal longitudinal diameter of the breast mass, and the acute angle between the two lines is the orientation angle of the breast mass (please refer to the Figs. 2–7 and Supplemental Materials S1). Before the initial acquisition of the orientation angle of breast mass, the two ultrasound physicians had discussed the protocol for standardized measurement of the orientation angle. To assess the reproducibility of the measurement, the orientation angle of the breast mass was measured twice in each of the 30 consecutively referred patients undergoing breast mass ultrasound examination at the start of the study. The agreement in determining parallel and nonparallel orientations of the breast masses based on visual assessment and quantitative measurement between the two ultrasound examiners was evaluated simultaneously.

### BI-RADS categorization for breast masses

The malignancy risk of the breast masses was evaluated with reference to the ultrasound BI-RADS. Histopathological diagnosis was used as standard reference. The lexicon descriptors for malignant breast masses included irregular shape, nonparallel orientation (orientation angle), ambiguous/lobulated/spiculated margin, microcalcifications, posterior shadowing, and edema (peripheral high echoic halo of the mass). Based on these lexicons, breast masses were classified into category 3 (no suspicious malignant feature), category 4a (one malignant feature), category 4b (two malignant features), category 4c (three malignant features), and category 5 (four malignant features and/or distant metastasis). Additional information such as age > 40 years, family history of breast cancer, nipple discharge, *etc.*, were also considered. The six ultrasound features of the breast mass and additional information were independently analyzed by two ultrasound physicians with over 16 years of experience. In cases of challenging interpretation, a consensus was reached through discussion between the two ultrasound physicians.

### Statistical analysis

Continuous variables following a normal distribution determined by Kolmogorov–Smirnov test were presented as mean ± standard deviation (M ± SD), and group comparisons were conducted using the independent samples *t*-test. Continuous variables not following a normal distribution were presented as median (interquartile range), and group comparisons were made using the Mann–Whitney U test. Count variables were expressed as the number of cases (percentage), and group comparisons were performed using the chi-square (*χ*^2^) test. The Cohen’s Kappa test was utilized to assess the agreement of categories of parallel and nonparallel orientations of breast masses based on visual assessment by the two ultrasound physicians, as well as the agreement of measurement of the orientation angle by the two ultrasound physicians. The intra-class correlation coefficient (ICC) was employed to evaluate the consistency of measurements of the orientation angle across three measurements by the same physician. The McNemar test was used to compare the differences between the categories of parallel and nonparallel orientations of breast masses based on visual assessment and the categories of orientation angle derived from ultrasound measurements. Spearman’s rank correlation analysis was conducted to investigate the correlation between preoperative data and the benign or malignant nature of breast masses. Receiver operating characteristic (ROC) curves were plotted using the measurements of orientation angle, and the cutoff value of orientation angle for breast mass malignancy assessment was determined according to the Youden index (sensitivity + specificity – 1). ROC curves were plotted for the analyses using different descriptors of nonparallel orientation and orientation angle, as well as the BI-RADS classification with and without incorporating the orientation angle. The area under the receiver operating characteristic (ROC) curves (AUCs) were calculated and compared, with comparisons between AUCs performed using the Z test. All statistical analyses were conducted using MedCalc 15.8.7 software. A two-sided *p*-value < 0.05 was considered significant.

## Results

### Final sample of the study

A total of 9,036 patients underwent breast ultrasound examination, out of which 6,893 patients were found to have breast masses. Among these, 954 of the 6,893 patients underwent core needle biopsy (253 masses) and/or surgical treatment (202 masses) and obtained pathological results. Ultimately, 253 patients with 253 breast masses were included in the study, with ages ranging from 18 to 74 years and a mean age of 43.88 ± 12.56 years. The maximum diameter of the breast masses ranged from 5.70 to 47.80 mm, with a mean of 20.38 ± 8.77 mm. Based on the histopathological diagnosis, 253 breast masses in 253 patients consisted of 134 benign masses and 119 malignant masses. Further details are provided in Fig. 2 and Table 1.

**Table 1:** Comparison of general conditions between two groups of patients.

Characteristic	Malignant nodule (*n* = 119)		Benign nodule (*n* = 134)	*P* value	
Age (y)	51.29 ± 10.07 (31–74) ^*^		37.30 ± 10.30 (18–67) ^*^	0.001	
BMI (kg/m^2^)	23.23 (21.64, 25.45) ^a^		22.03 (19.78,23.83) ^a^	0.001	
Family history of breast cancer			0.005	
Yes	11 (9.2)		2 (1.5)	
No	108 (90.8)		132 (98.5)	
Palpable mass			<0.001	
Yes	103 (86.6)		78 (58.2)	
No	16 (13.4)		56 (41.8)	
Breast pain			0.807	
Yes	26 (21.8)		31 (23.1)	
No	93 (78.2)		103 (76.9)	
Nipple discharge			0.864	
Yes	4 (3.8)		4 (3.0)	
No	115 (96.6)		130 (97.0)	
Skin edema			0.354	
Yes	5 (4.2)		2 (1.5)	
No	114 (95.8)		132 (98.5)	
Position of mass			0.233	
Left	64 (53.8)		62 (46.3)	
Right	55 (46.2)		72 (53.7)	
Quadrant				0.093	
Upper outer	60 (50.4)	62 (46.3)	
Upper inner	20 (16.8)	26 (19.4)	
Lower inner	8 (6.7)	21 (15.7)	
Lower outer	31 (26.1)	25 (18.7)	
Enlarged lymph nodes in axillary regions			<0.001
Yes	31 (26.1)		8 (6.0)	
No	88 (73.9)		126 (94.0)	
Maximal diameter (mm)	20.50 (14.55, 25.85)^a^		17.45 (13.50, 25.10)^a^	0.190	
Orientation angle (^∘^)			<0.001	
≤ 22.3	20 (16.8)		112 (83.6)	
> 22.3	99 (83.2)		22 (16.4)	

**Notes.**

Unless otherwise indicated, data are counts with percentages in parentheses.

* mean data are ± standard deviation, Data in parentheses are ranges.

a median, Data in parentheses are 25th percentile and 75th percentile.

BMI Body mass index

### Outcomes of statistical analyses

**Figure 8:**
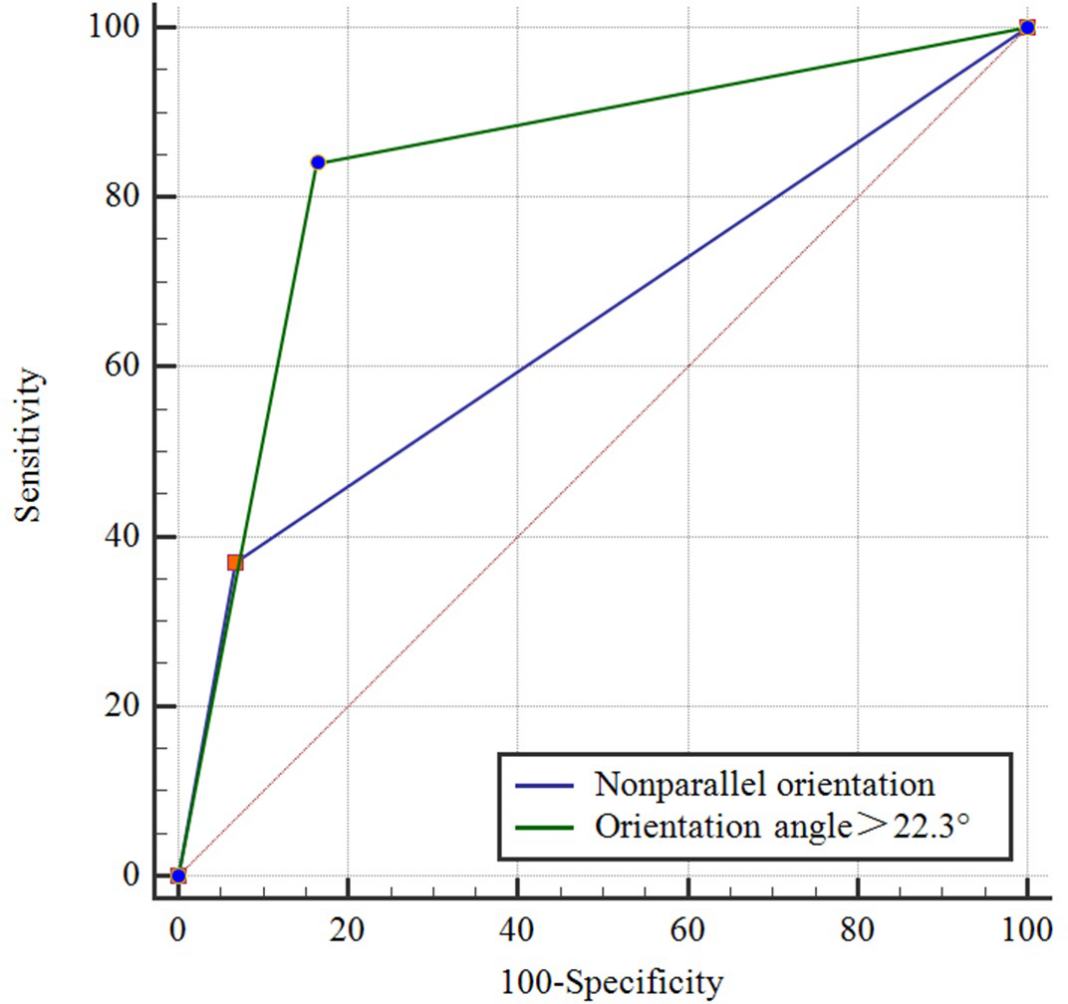
Comparison of receiver operating characteristic curves using descriptor of parallel/nonparallel orientation of Breast Imaging Reporting and Data System and orientation angle as alternative descriptor for malignancy assessment.

**Figure 9:**
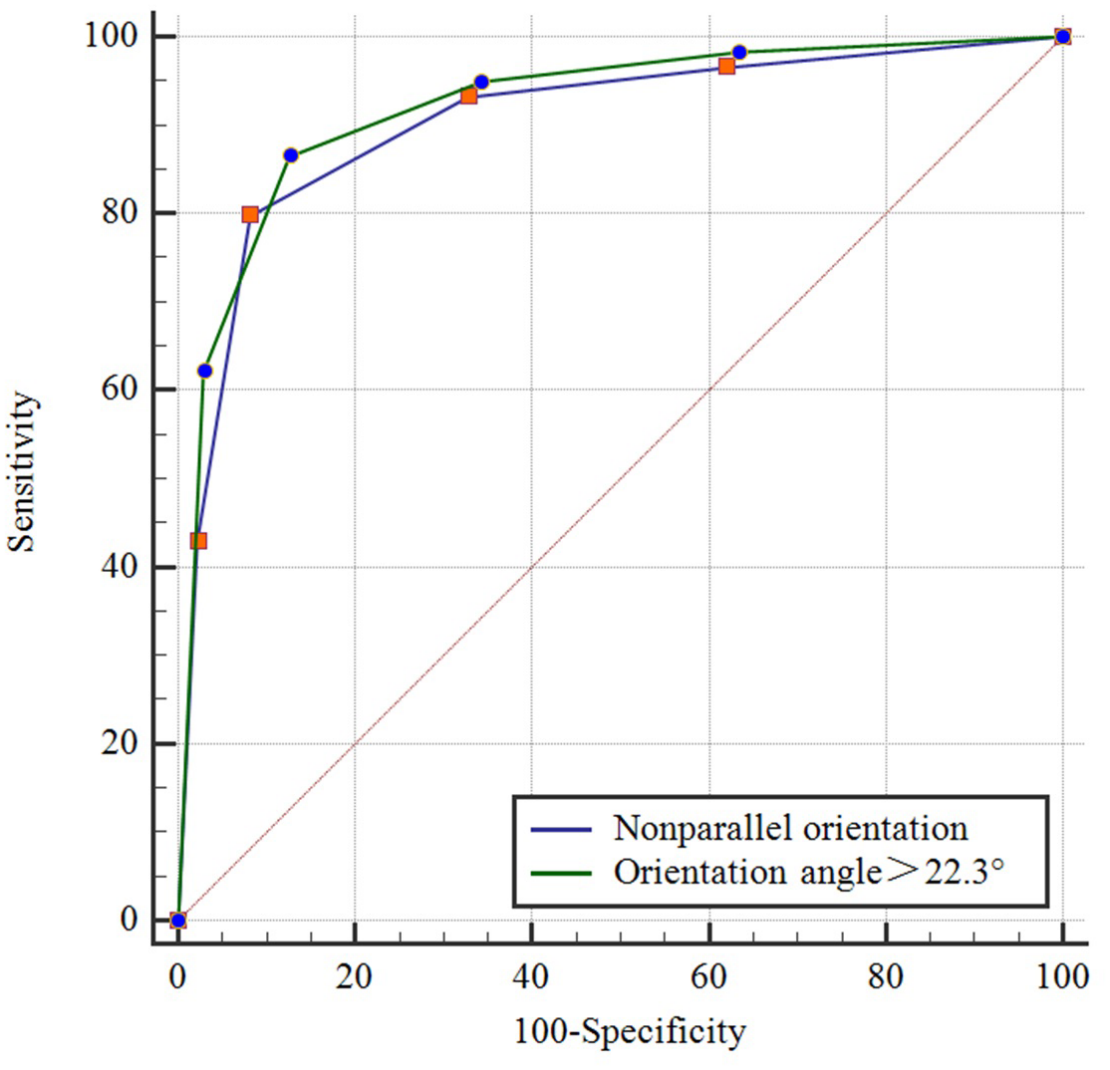
Comparison of receiver operating characteristic curves using conventional descriptor of parallel/nonparallel orientation of Breast Imaging Reporting and Data System and a combination of them for malignancy assessment of breast masses.

Comparisons of the demographic characteristics and general conditions of patients with benign and malignant breast masses are listed in Table 1. Figures 2, 3, 4, 5, 6 and 7 are representative images of the measurement of orientation angle of benign and malignant breast masses, and their categories as nonparallel orientation based on visual assessment and quantitative measurement. The Kappa value of the agreement in determining nonparallel orientation of the breast masses based on visual assessment between two ultrasound examiners was 0.531 (*p* < 0.001). The Kappa value of the measurement of the orientation angle between two ultrasound physicians was 0.858 (*p* < 0.001). The ICC for the measurement of the orientation angle was 0.975 (*p* < 0.001). The Youden index was 0.6771, the cutoff of orientation angle of breast masses for breast malignancy was > 22.3°, and the sensitivity and specificity were 84.87% and 82.84%, respectively. Significant differences were observed between the categories of parallel and nonparallel orientations of the breast masses based on visual assessment and the categories of orientation angle >22.3° and orientation angle ≤ 22.3° of the breast masses (*p* < 0.001). Spearman’s rank correlation analysis indicated significant positive correlations of the patient’s age (*r* = 0.550, *p* < 0.001), BMI (*r* = 0.234, *p* < 0.001), family history (*r* = 0.175, *p* = 0.005), palpable mass (*r* = 0.314, *p* < 0.001), abnormal axillary lymph nodes (*r* = 0.278, *p* < 0.001), and the measurement of the orientation angle (*r* = 0.667, *p* < 0.001) with the pathological malignancy of breast masses. Forty six breast masses categorized BI-RADS 3 (*n* = 4), 4a (*n* = 4), 4b (*n* = 14), and 4c (*n* = 24) based on visual assessment of nonparallel orientation were upgraded correspondingly as category BI-RADS 4a, 4b, 4c, and 5 after using orientation angle, and there was no breast mass being downgraded. The AUCs for using the criteria of nonparallel orientations of the breast masses based on visual assessment and using the cutoff of orientation angle > 22.3° of the breast masses were 0.651 and 0.838, respectively, with a significant difference between them (*p* < 0.001); details are provided in Table 2 and Fig. 8. The AUCs for BI-RADS classifications before and after incorporating the orientation angle > 22.3° were 0.905 and 0.922, respectively, with a significant difference (*p* = 0.024). The optimal cutoff value for differentiating benign from malignant breast masses, as determined by the Youden index (sensitivity + specificity − 1), was > 4b for BI-RADS with and without incorporating the orientation angle > 22.3°; details are shown in Table 3 and Fig. 9.

**Table 2:** Comparison of the diagnostic performance between nonparallel orientation and orientation angle of breast mass for the malignancy evaluation.

Item	Sens (%)	Spec (%)	PPV (%)	NPV (%)	AUC (95% CI)	OR
Nonparallel orientation	36.97	93.28	83.02	62.50	0.651 (0.589–0.71)	8.15
Orientation angle >22.3°	84.03	83.58	81.82	84.85	0.838 (0.787–0.88)	25.20
*P* value	<0.001	0.013	0.849	<0.001	<0.001	

**Notes.**

Sens sensitivity Spec specificity PPV Positive Predictive Value NPV Negative Predictive Value AUC Area Under the Curve 95% CI 95% Confidence Interval OR Odds Ratio

**Table 3:** Comparison of the diagnostic performance between using nonparallel orientation and orientation angle for BI-RADS categorization of breast masses.

Item	Sens (%)	Spec (%)	PPV (%)	NPV (%)	AUC (95% CI)	OR	Cutoff value
Nonparallel orientation	79.83	91.79	89.62	83.67	0.905 (0.862∼0.938)	44.26	BI-RADS>4b
Orientation angle >22.3°	86.55	87.31	85.83	87.97	0.922 (0.881∼0.952)	44.31	BI-RADS>4b
*P* value	0.166	0.231	0.388	0.305	0.024		

**Notes.**

BI-RADS Breast Imaging Reporting and Data System Sens sensitivity Spec specificity PPV Positive Predictive Value NPV Negative Predictive Value AUC Area Under the Curve 95% CI 95% Confidence Interval OR Odds Ratio

## Discussion

The BI-RADS ultrasound lexicon describes nonparallel orientation as an important feature associated with the malignancy risk of breast masses (D’Orsi et al., 2013). Studies by Gu et al. (2022) and Guo et al. (2018) have found that malignant breast masses are more likely to exhibit the nonparallel orientation feature than benign breast masses. Additionally, Cao et al. (2014) and Wang et al. (2020) have found that invasive breast cancers with a nonparallel orientation feature are associated with a worse prognosis. Therefore, it is crucial to pay more attention to the nonparallel orientation feature of breast masses during breast mass evaluation.

In this study, the Kappa value of two physicians assessing the nonparallel orientation features of breast masses by visual assessment was 0.531. In contrast, the Kappa value of two physicians assessing the orientation angle of breast masses derived from ultrasound quantitative measurement was 0.858, indicating excellent agreement. This value was significantly higher than the previous moderate agreement observed with visual assessment. This suggests that variables obtained through ultrasound quantitative measurement are more reliable and objective than those obtained through visual assessment. The nonparallel orientation feature obtained through visual assessment for assessing the malignancy risk of breast masses in this study had a sensitivity of 36.97%, specificity of 93.28%, and an AUC of 0.651. These findings were consistent with a previous study that reported a sensitivity of 56.3%, specificity of 88.0%, and accuracy of 70.2% (Badu-Peprah et al., 2024). The ICC of 0.975 for the ultrasound quantitative measurement of the orientation of breast masses in this study demonstrates excellent reproducibility and consistency. Using orientation angle as an alternative descriptor of ultrasound BI-RADS for the malignancy assessment of breast masses upgraded BI-RADS category, and resulted in a sensitivity of 84.03%, specificity of 83.58%, and an AUC of 0.838. The specificity and AUC were higher than those reported in previous studies (Yoon et al., 2016; Badu-Peprah et al., 2024). There was a significant difference in the AUCs of breast malignancy risk assessment between using the nonparallel orientation based on visual assessment and the orientation angle > 22.3° derived from ultrasound quantitative measurement (*p* < 0.001). The McNemar test indicated a significant difference in the assessment of orientation between the two methods (*p* < 0.001). Therefore, the ultrasound quantitative assessment of breast mass orientation has significantly higher diagnostic efficacy than visual assessment based assessment, which is consistent with previous findings (Chen & Wu, 2024).

In this study, when the orientation angle >22.3° was not incorporated into the ultrasound BI-RADS for the malignancy risk assessment of breast masses, it exhibited a sensitivity of 79.83%, specificity of 91.79%, and an AUC of 0.905, which aligns with findings from a previous study (Gu et al., 2022). When the orientation angle > 22.3° was integrated into the ultrasound BI-RADS for the malignancy risk assessment of breast masses, it showed a sensitivity of 86.55%, specificity of 87.31%, and an AUC of 0.922, indicating higher diagnostic efficacy compared to previous studies (Gu et al., 2022; Guo et al., 2018). Additionally, this study results were very close to the results obtained in the study by Chen & Wu (2024) that a sensitivity and specificity of 88.5% and 86.2%, respectively, when using the orientation angle > 22.9° as a cutoff. The difference in AUCs between ultrasound BI-RADS with and without incorporating the orientation angle > 22.3° was significant (*p* = 0.024). These results suggest that using orientation angle > 22.3° as an alternative for the descriptor of nonparallel orientation for the evaluation of malignancy risk of breast masses within ultrasound BI-RADS can enhance diagnostic efficacy.

A stronger positive correlation was found between the orientation angle > 22.3° and pathological malignancy in this study (*r* = 0.667, *p* < 0.001), indicating that a larger orientation angle is associated with a higher likelihood of malignancy. Literally, parallel orientation should have a zero orientation angle, and nonparallel orientation with orientation angle is larger than zero, but the results of this study showed that not all orientation angle larger than zero is nonparallel orientation, and some of them belong to parallel orientation based on visual assessment. Therefore, ultrasound quantitative measurement of breast mass orientation provides a quantitative standard for clinical practice, enabling the description more objective, scientific, and accurate.

In this study, the ultrasound BI-RADS incorporating both descriptors of nonparallel orientation and orientation angle > 22.3° had a cutoff value of ultrasound BI-RADS category > 4b for differentiating benign from malignant breast masses. This indicates that incorporating the orientation angle > 22.3° into BI-RADS did not change the cutoff value. Which is consistent with the previous study (Chen & Wu, 2024). We believe the reason for this is that the ultrasound BI-RADS category 4b encompasses a wide range of likelihood of malignancy risk, from 10% to no more than 50%. When using nonparallel orientation for evaluation, the cutoff may tend to be at the lower limit, and similarly, when using the orientation angle > 22.3° for evaluation, the cutoff may tend to be at the upper limit.

The orientation angle of breast mass larger than 22.3° is equivalent to nonparallel orientation in this study, which is not the same as that in the previous study that the orientation angle larger than 22.9°, and there is a marginal difference. The researchers believe it is the result of systematic and accidental errors of quantitative measurement, and it does not impact the value of orientation angle as a useful complementary descriptor for nonparallel orientation. The orientation angle may be a limited range, other than a specific value. When a breast mass presents conspicuous nonparallel orientation, quantitative measurement is not required. On the other hand, when the parallel or nonparallel orientation of a breast mass is hard to determine by visual assessment, quantitative measurement of orientation angle can solve the challenge. These have been validated in this study that there were 46 breast masses categorized ultrasound BI-RADS 3, 4a, 4b, and 4c based on visual assessment of nonparallel orientation were upgraded correspondingly as category ultrasound BI-RADS 4a, 4b, 4c, and 5 after using orientation angle, and there was no breast mass being downgraded.

This study acknowledges two primary limitations. This study was conducted at a single center with a relatively small sample size. Future studies should incorporate larger, multi-center cohorts to validate these findings. Second, despite the inclusion of more benign (*n* = 134) than malignant (*n* = 119) masses, the exclusion of a substantial number of BI-RADS category 3 lesions without histopathological confirmation may have introduced spectrum and verification biases, This could have led to an overestimation of diagnostic performance. Third, a technical limitation exists: the orientation angle measurements were performed using two specific ultrasound systems, while the broader study population was imaged with a variety of systems. The feasibility and consistency of this quantitative measurement across different ultrasound systems require further validation.

## Conclusions

The quantitative orientation angle provides a more reproducible and objective assessment of breast mass orientation than subjective visual assessment and description. In cases where visual determination is ambiguous, this quantitative measure can aid in decision-making. Incorporating the orientation angle into multi-descriptor ultrasound BI-RADS assessment of breast masses allows for a more precise categorization than the conventional descriptor alone, thereby improving overall diagnostic performance. This orientation angle may be further validated and has the potential to be used as a complementary descriptor for orientation within the ultrasound BI-RADS.

##  Supplemental Information

10.7717/peerj.20992/supp-1 Supplemental Information 1 Protocol for Measuring the Orientation Angle of a Breast Mass on Ultrasonography

10.7717/peerj.20992/supp-2 Supplemental Information 2 Raw data of measurement of the orientation angle of the breast mass

10.7717/peerj.20992/supp-3 Supplemental Information 3 STARD checklist

## Additional Information and Declarations

### Data Availability

The following information was supplied regarding data availability: The raw data is available in the Supplemental File.
