# Supplementary material for: Quantitative outperforms visual assessment of nonparallel orientation in ultrasound breast imaging reporting and data system
Source: PeerJ. 2026 Mar 18;14:e20992. doi: 10.7717/peerj.20992 (PMC13005611; doi:10.7717/peerj.20992)
Supplement: Supplemental Information 1 [file peerj-14-20992-s001.pdf]

Quantitative Outperforms Visual Assessment of Parallel/Non-parallel Orientation in Breast Ultrasound BI-RADS

Kailiang Chen[1,2], Qingfang Chen[1], Size Wu [Corresp. 1]

[1] Department of Ultrasound, The First Affiliated Hospital of Hainan Medical University, No.31, Longhua Road, Haikou570102, China.

[2]Class 2025 of Rehabilitation, UWE College of Hainan Medical University, No.3, Xueyuan Road, Haikou571199, China.

Protocol for Measuring the Orientation Angle of a Breast Mass on Ultrasonography

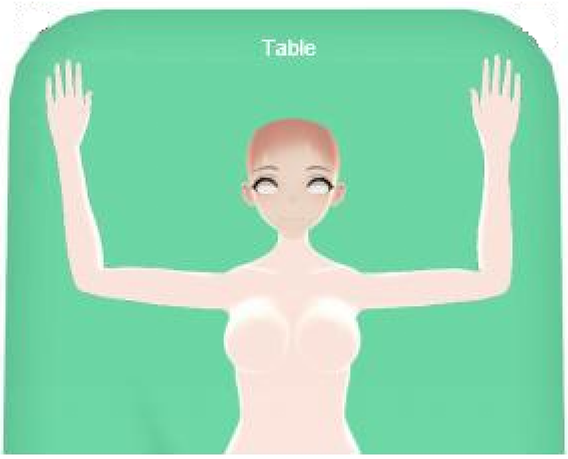

Figure 1: Patient positioning for breast ultrasound examination. The patient lies supine without a head pillow, with arms abducted. The upper arms are perpendicular to the torso, and the forearms are perpendicular to the upper arms.

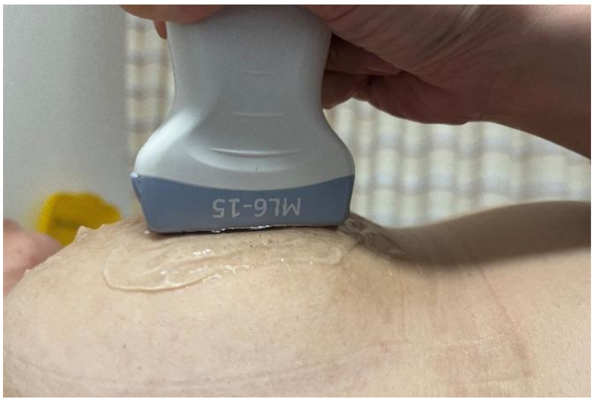

Figure 2a: Scanning technique. After applying adequate coupling gel, the breast is scanned for abnormalities using a high-frequency linear transducer (6-15 MHz). The operator holds the transducer vertically to the skin without compressing the breast.

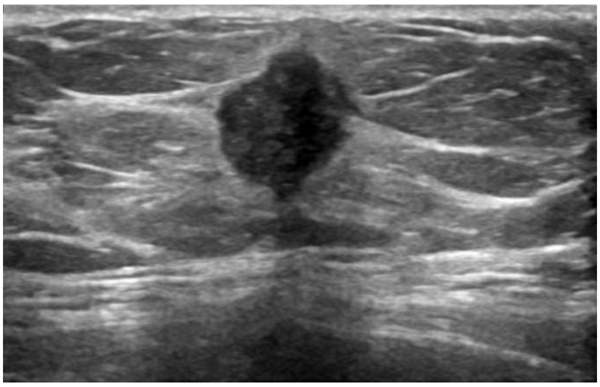

Figure 2b: Ultrasound image of a mass in the left breast of a 48-year-old woman. The nodule exhibits an irregular shape, solid composition, hypoechoic echogenicity, spiculated margin, and absence of posterior acoustic features.

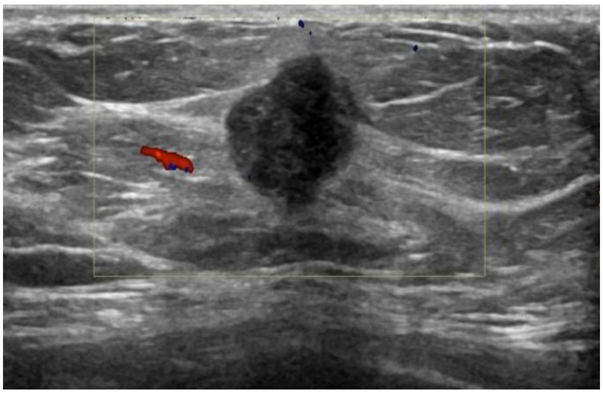

Figure 2c: Color Doppler flow imaging demonstrates no detectable vascularity within the breast mass.

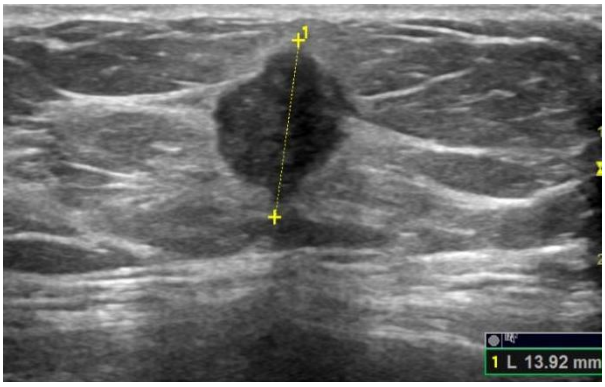

Figure 2d: Measurement of the mass in one dimension. The maximal diameter obtained in this plane is 13.92 mm.

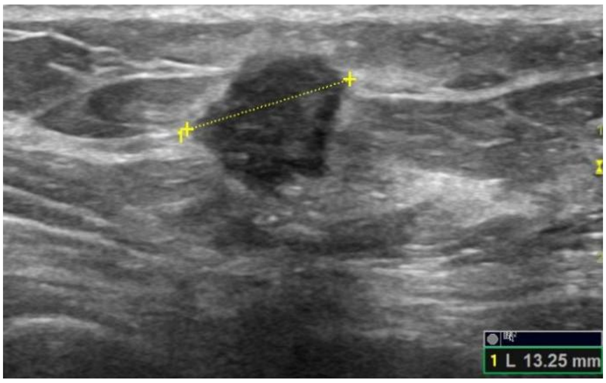

Figure 2e: Measurement of the mass in a second dimension. The maximal diameter obtained in this plane is 13.25 mm.

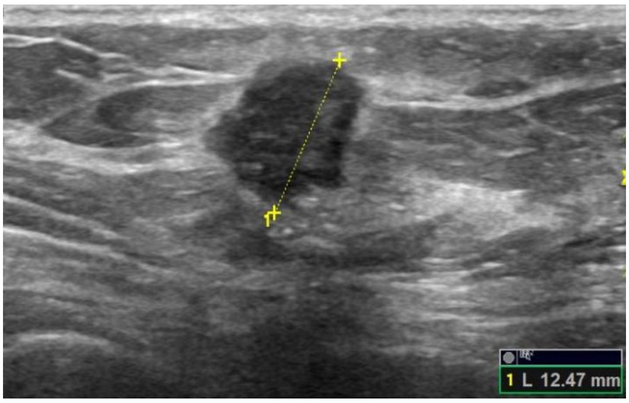

Figure 2f: Measurement of the mass in a third dimension. The maximal diameter obtained in this plane is 12.47 mm.

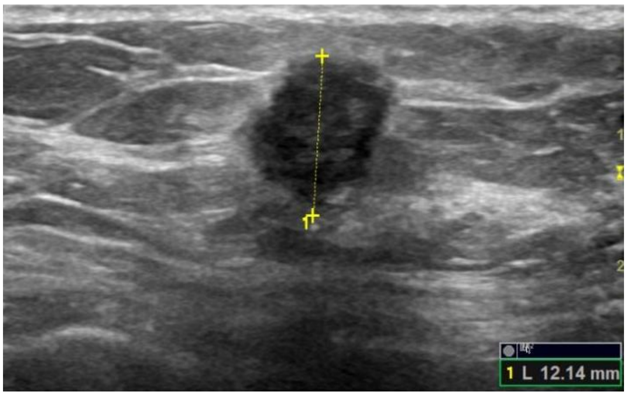

Figure 2g: Measurement of the mass in a fourth dimension. The maximal diameter obtained in this plane is 12.14 mm.

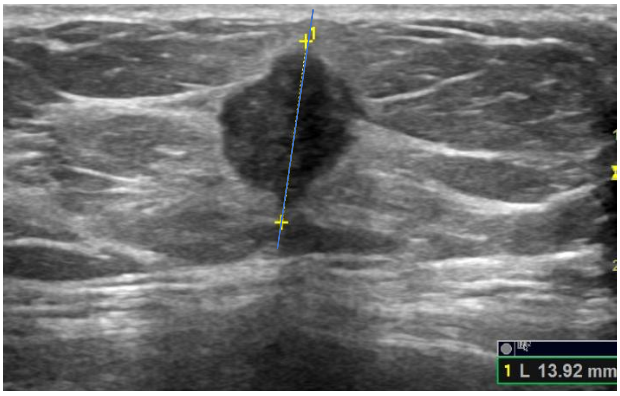

Figure 2h: Identification of the maximal dimension. Review of all measurements (Figures 2d-g) identifies the maximal diameter (13.92 mm) and its corresponding dimension. The orientation angle is measured on this maximal dimension by extending the line representing the maximal diameter towards the skin surface.

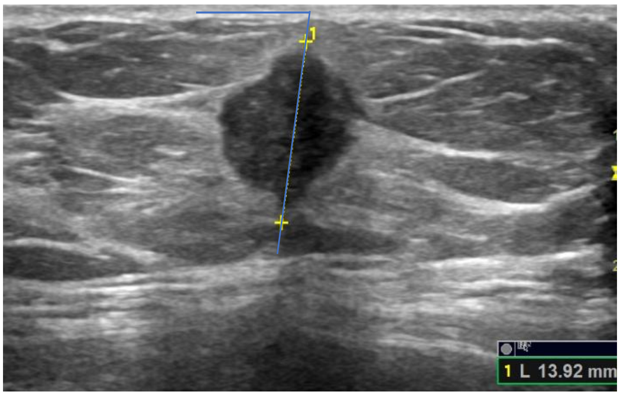

Figure 2i: Construction of the reference line. A line parallel to the skin surface is drawn to intersect the extended line of the maximal diameter of the maximal dimension of the breast mass.

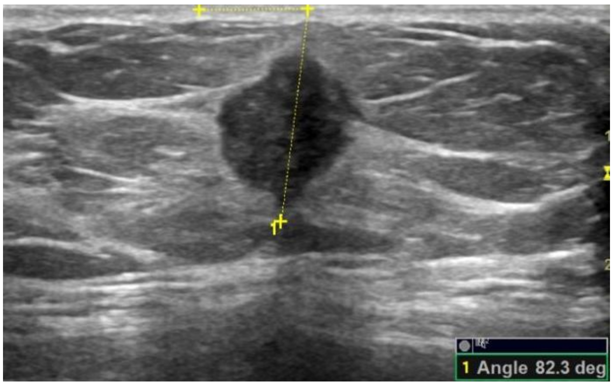

Figure 2j: Angle measurement. The acute angle (82.3°) between the skin-parallel line and the extended line of the maximal diameter of the maximal dimension of the breast nodule is measured using the ultrasound system's built-in software. This angle defines the orientation angle of the breast nodule.
